# NSD Histone Methyltransferases in Solid Tumors: Biological Functions, Oncogenic Mechanisms and Therapeutic Targeting

**DOI:** 10.3390/ijms27188115

**Published:** 2026-09-12

**Authors:** Yan-ting Yann Zhang, Andrew Plenn, Joey Sun, Cun Zhang, Phil Evans, Junsong Zhao, Huicong Li, Aurpita Shaha, Xiaodan Yao, Haiying Chen, Francis Spitz, Generosa Grana, Jia Zhou, Shumei Song

**Affiliations:** 1 Coriell Institute for Medical Research, 403 Haddon Ave, Camden, NJ 08103, USAphiljordan46@gmail.com (P.E.);; 2 Department of Biomedical Sciences, Cooper Medical School of Rowan University, 401 Broadway, Camden, NJ 08103, USA; 3 Camden Cancer Research Center, 403 Haddon Ave, Camden, NJ 08103, USA; 4 Department of Pharmacology and Toxicology, University of Texas Medical Branch, Basic Science Building, 301 University Boulevard, Galveston, TX 77555, USAjizhou@utmb.edu (J.Z.); 5 MD Anderson Cancer Center at Cooper, Cooper University Hospital, 2 Cooper Plaza, Camden, NJ 08103, USA; 6 Cooper University Hospital, Department of Surgery, Cooper Medical School of Rowan University, 1 Cooper Plaza, Camden, NJ 08103, USA

**Keywords:** NSD1, NSD2, NSD3, epigenetics, histone methylation, solid tumors, targeted therapy

## Abstract

Epigenetic dysregulation is a defining feature of solid tumors. Among epigenetic regulators, the nuclear receptor-binding SET domain (NSD) family of histone methyltransferases, comprising NSD1, NSD2, and NSD3, have emerged as critical mediators of oncogenic chromatin remodeling and transcriptional regulation. These enzymes primarily catalyze histone H3 lysine 36 (H3K36) methylation, thereby regulating chromatin accessibility, transcriptional programs, DNA damage repair, and genome stability. Aberrant expression, mutation, amplification, and chromosomal rearrangement of NSD family members have been identified across tumors, establishing them as key epigenetic drivers of malignancy. Accumulating evidence demonstrates that NSD proteins promote multiple hallmarks of cancer, including sustained proliferative signaling, invasion and metastasis, immune evasion, metabolic reprogramming, genome instability, and therapeutic resistance. Although NSD proteins share overlapping catalytic functions, each exhibit distinct biological roles and mechanisms of dysregulation in solid tumors. Advances in structural biology, medicinal chemistry, and targeted protein degradation have accelerated the development of selective NSD inhibitors, chromatin-reader antagonists, and proteolysis-targeting chimeras (PROTACs), establishing the feasibility of pharmacologically targeting NSD-dependent epigenetic pathways. In this review, we summarize the biological functions of the NSD family, discuss their oncogenic mechanisms in solid tumors, and highlight recent progress in therapeutic targeting.

## 1. Introduction

Epigenetic dysregulation is now recognized as a fundamental hallmark of cancer, complementing genetic alterations in driving malignant transformation, tumor progression, and therapeutic resistance. Among the diverse epigenetic regulators implicated in cancer, histone lysine methyltransferases have emerged as critical determinants of chromatin organization and transcriptional control. The nuclear receptor-binding SET domain (NSD) family of histone methyltransferases, comprising NSD1, NSD2 (WHSC1/MMSET), and NSD3 (WHSC1L1), represent a highly conserved group of chromatin regulators that primarily catalyze mono- and dimethylation of histone H3 lysine 36 (H3K36me1/2). These modifications are generally associated with transcriptionally active chromatin and play essential roles in regulating gene expression, enhancer activity, DNA damage repair, cellular differentiation, and genome stability. By coordinating chromatin accessibility with transcriptional output, NSD proteins orchestrate biological processes that are indispensable for normal development and tissue homeostasis.

Over the past two decades, accumulating evidence has established the NSD family as a major class of epigenetic drivers in human malignancies. Recurrent mutations, gene amplifications, chromosomal translocations, and aberrant expression of NSD family members have been identified across both hematologic and solid tumors, resulting in widespread chromatin remodeling and activation of oncogenic transcriptional networks [1]. Although the oncogenic role of NSD2 was first recognized through the t(4;14) translocation in multiple myeloma [2], subsequent studies have demonstrated that all three NSD proteins contribute to tumorigenesis through distinct but often overlapping mechanisms in solid tumors. Depending on cellular context, NSD proteins regulate proliferation, epithelial–mesenchymal transition, metastasis, metabolic adaptation, immune evasion, and resistance to chemotherapy, endocrine therapy, targeted agents, and immunotherapy. Increasing studies have further highlighted the importance of NSD-mediated epigenetic regulation in solid tumors, including breast cancer, prostate cancer, lung cancer and gastrointestinal (GI) malignancies. In these cancers, altered NSD activity reshapes chromatin landscapes, reprograms enhancer activity, and cooperates with lineage-specific transcription factors and oncogenic signaling pathways to promote tumor initiation, progression, and metastatic dissemination. Beyond their intrinsic effects on tumor cells, NSD proteins also influence the tumor microenvironment by regulating immune cell infiltration, inflammatory signaling, and tumor plasticity, underscoring their multifaceted roles in cancer biology.

Historically, NSD proteins were considered difficult therapeutic targets because of their complex multidomain architecture, autoinhibitory mechanisms, and the absence of readily druggable catalytic pockets [3]. However, recent advances in structural biology, cryo-electron microscopy, fragment-based drug discovery, and chemical biology have substantially improved our understanding of NSD regulation and enabled the development of selective catalytic inhibitors, PWWP domain antagonists, and targeted protein degraders [4,5]. More recently, highly potent clinical-grade NSD2 inhibitors have demonstrated anti-tumor activity in preclinical models of solid tumors [6]. These advances have transformed the NSD family from a group of challenging chromatin regulators into an emerging class of therapeutically actionable epigenetic targets.

In this review, we summarize current knowledge of the biological functions of the NSD family, discuss the molecular mechanisms by which NSD proteins drive solid tumor progression, and highlight recent advances in therapeutic strategies targeting NSD-dependent epigenetic pathways.

## 2. Biological Functions of NSD Proteins

The nuclear receptor-binding SET domain (NSD) family comprises a group of evolutionarily conserved histone methyltransferases that function as critical regulators of chromatin architecture and gene expression. The family consists of three members, NSD1, NSD2, and NSD3, which share a highly conserved multidomain structure while exhibiting distinct biological functions and tissue-specific roles. Since their initial discovery, NSD proteins have emerged as central mediators of epigenetic regulation, influencing cellular differentiation, developmental patterning, DNA damage responses, transcriptional control, and oncogenic transformation. The growing recognition of their involvement in human disease has positioned the NSD family at the intersection of chromatin biology and cancer research. NSD1 was the first family member identified and was initially characterized as a nuclear receptor-interacting protein capable of functioning as both a transcriptional coactivator and a corepressor. Early studies demonstrated that NSD1 contains multiple nuclear receptor interaction domains and participates in hormone receptor-mediated transcriptional regulation, establishing its role as a chromatin-associated transcriptional regulator [7]. Subsequent investigations revealed that NSD1 possesses intrinsic histone methyltransferase activity and belongs to a broader family of SET domain-containing proteins involved in chromatin modification and transcriptional control. The discovery of NSD2 followed shortly thereafter through studies of the t(4;14) chromosomal translocation in multiple myeloma, which identified the Multiple Myeloma SET Domain (MMSET) gene as a recurrent target of genomic rearrangement [2]. NSD3 was subsequently identified as WHSC1L1, a gene closely related to NSD1 and NSD2 that share extensive structural homology and chromatin regulatory functions [8]. Collectively, these discoveries established the NSD family as a distinct group of chromatin-modifying enzymes with important biological and pathological functions.

**Figure 1:**
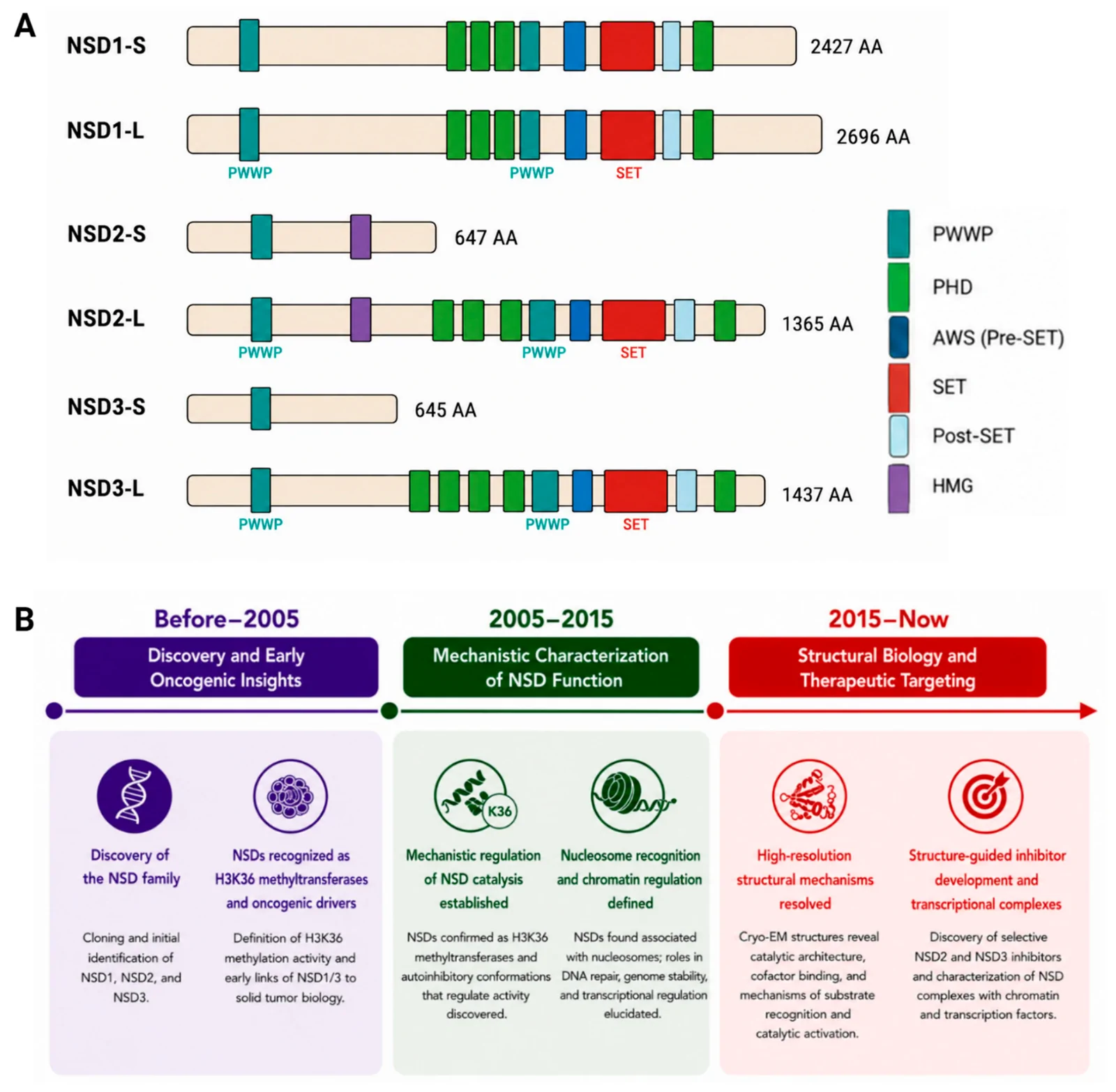
Conserved domain architecture (**A**) and research progress timeline (**B**) of NSD proteins in solid tumor. Created in BioRender. Plenn, A. (2026) https://BioRender.com/9gg4jkl (accessed on 30 August 2026).

Although individual NSD family members exhibit unique biological activities, all three proteins share a conserved multidomain architecture that underlies their ability to regulate chromatin structure and gene expression. Central to their function is the catalytic SET domain, which mediates lysine methyltransferase activity. Surrounding the SET domain are multiple chromatin-recognition motifs, including plant homeodomain (PHD) zinc fingers and proline-tryptophan-tryptophan-proline (PWWP) domains, which facilitate interactions with chromatin and contribute to genomic targeting. NSD proteins also contain a SET-associated Cys-rich (SAC) domain that stabilizes catalytic activity, as well as additional regulatory motifs including high mobility group (HMG) domains and nuclear receptor interaction motifs. The coordinated activity of these domains allows NSD proteins to recognize chromatin, interact with transcriptional machinery, and modify histone substrates in a highly regulated manner (Figure 1A). The primary enzymatic function of NSD proteins involves methylation of histone H3 lysine 36 (H3K36), a chromatin mark generally associated with transcriptionally active regions of the genome. Early biochemical studies established that NSD family members function as H3K36 methyltransferases and contribute to gene expression regulation through the deposition of mono- and dimethylated H3K36 marks [9]. Subsequent investigations demonstrated that NSD2 serves as a major source of H3K36me2 in mammalian cells and that alterations in NSD activity profoundly influence chromatin organization and transcriptional output [10]. The biological significance of H3K36 methylation extends beyond transcriptional activation alone. NSD-mediated deposition of H3K36me2 antagonizes the activity of Polycomb repressive complexes and limits accumulation of H3K27me3, thereby establishing a dynamic balance between active and repressive chromatin states. Through this mechanism, NSD proteins regulate accessibility of regulatory elements, enhancer activity, and transcription factor recruitment across large genomic regions. A major advance in understanding NSD biology emerged from structural studies investigating how these enzymes engage nucleosomal substrates. Initial biochemical analyses suggested that NSD proteins exist in an autoinhibited conformation in which access to the catalytic SET domain is restricted [3]. This model was subsequently confirmed through structural studies demonstrating that intramolecular interactions within NSD proteins prevent inappropriate methyltransferase activity and maintain catalytic regulation [4]. Cryo-electron microscopy studies revealed that nucleosome binding induces conformational rearrangements that relieve autoinhibition and permit productive engagement of histone substrates [4,11]. These findings transformed the understanding of NSD function by demonstrating that catalytic activation is tightly coupled to chromatin recognition and nucleosome interaction. Rather than functioning as constitutively active enzymes, NSD proteins operate through highly regulated mechanisms that integrate chromatin context with enzymatic activity. Beyond direct histone modification, NSD proteins exert broad effects on transcriptional regulation. H3K36 methylation promotes recruitment of transcriptional machinery and facilitates efficient transcriptional elongation by RNA polymerase II. NSD-mediated chromatin remodeling influences accessibility of promoters and enhancers while coordinating interactions between transcription factors and co-regulatory complexes. Through these mechanisms, NSD proteins regulate the expression of genes involved in cellular proliferation, differentiation, metabolism, and stress responses. Importantly, NSD-dependent transcriptional regulation often occurs through large-scale chromatin reorganization rather than isolated gene-specific effects, allowing these proteins to coordinate complex transcriptional programs that influence cell fate decisions and developmental processes. 

Growing evidence indicates that NSD proteins perform functions extending beyond histone methylation [12]. Several studies have demonstrated that NSD family members participate in DNA damage repair pathways and maintenance of genomic stability [13]. H3K36 methylation provides an essential platform for recruiting DNA repair factors, and disruption of NSD activity compromises cellular responses to DNA damage. NSD proteins have also been implicated in the regulation of replication dynamics, chromatin restoration following repair, and maintenance of genome integrity under conditions of cellular stress. These observations suggest that NSD proteins function not only as transcriptional regulators but also as guardians of chromatin stability. In addition to histone substrates, NSD proteins have been reported to methylate non-histone proteins [14,15] and participate in protein interaction networks that influence transcriptional regulation independently of catalytic activity. Interactions with transcription factors, chromatin remodeling complexes, and signaling pathways enable NSD proteins to integrate epigenetic regulation with broader cellular signaling networks. These observations have challenged the traditional view of NSD proteins as simple histone methyltransferases and instead support their classification as multifunctional chromatin regulators. 

Functional diversity within the NSD family is further increased through alternative splicing and the generation of distinct protein isoforms. NSD1 produces both long and short isoforms that differ in domain organization and potentially in biological activity. Similarly, the NSD2 locus encodes multiple protein products, including full-length NSD2 (MMSET-II), the shorter MMSET-I isoform, and RE-IIBP. NSD3 generates three principal isoforms: NSD3-long (NSD3L), NSD3-short (NSD3S), and WHISTLE. These isoforms exhibit substantial differences in domain composition and catalytic capability, resulting in diverse biological functions. Full-length proteins generally retain the catalytic SET domain and function as chromatin-modifying enzymes, whereas shorter isoforms often lack methyltransferase activity but retain the ability to regulate transcription through protein–protein interactions and chromatin recruitment mechanisms. The biological significance of these isoforms has become increasingly apparent in both normal development and cancer, where distinct isoforms may exert complementary or even opposing effects on cellular behavior. The importance of NSD proteins in human disease is underscored by their frequent involvement in chromosomal translocations, amplifications, and recurrent genetic alterations. The t(4;14) translocation involving NSD2 is one of the best-characterized examples and has established a direct link between NSD dysregulation and malignant transformation [16]. Additional rearrangements involving NSD1 and NSD3, including NUP98-NSD1 [17] and NSD3-NUT [18] fusion proteins, further demonstrated the oncogenic potential of aberrant NSD activity. These observations provided some of the earliest evidence connecting epigenetic dysregulation to cancer development and stimulated extensive investigation into the role of NSD proteins in tumorigenesis.

Collectively, the available evidence establishes NSD proteins as central regulators of chromatin biology. Through coordinated control of H3K36 methylation, chromatin accessibility, transcriptional elongation, DNA damage responses, and genome stability, NSD family members influence a wide range of biological processes that are essential for normal cellular function. The discovery of their involvement in human malignancies has further highlighted the importance of NSD-mediated epigenetic regulation and laid the foundation for subsequent investigations into their roles in solid tumors and therapeutic targeting. We have organized the development of NSD cancer research into three major periods and highlighted the progression from the initial characterization of NSD proteins to the elucidation of their roles in cancer biology and the development of NSD-targeted therapeutic strategies (Figure 1B). Understanding the structural organization and biological functions of NSD proteins is therefore essential for appreciating how dysregulation of these enzymes contributes to cancer progression and why they have emerged as promising targets for therapeutic intervention. 

## 3. Oncogenic Mechanisms of NSD Proteins in Solid Tumor

**Figure 2:**
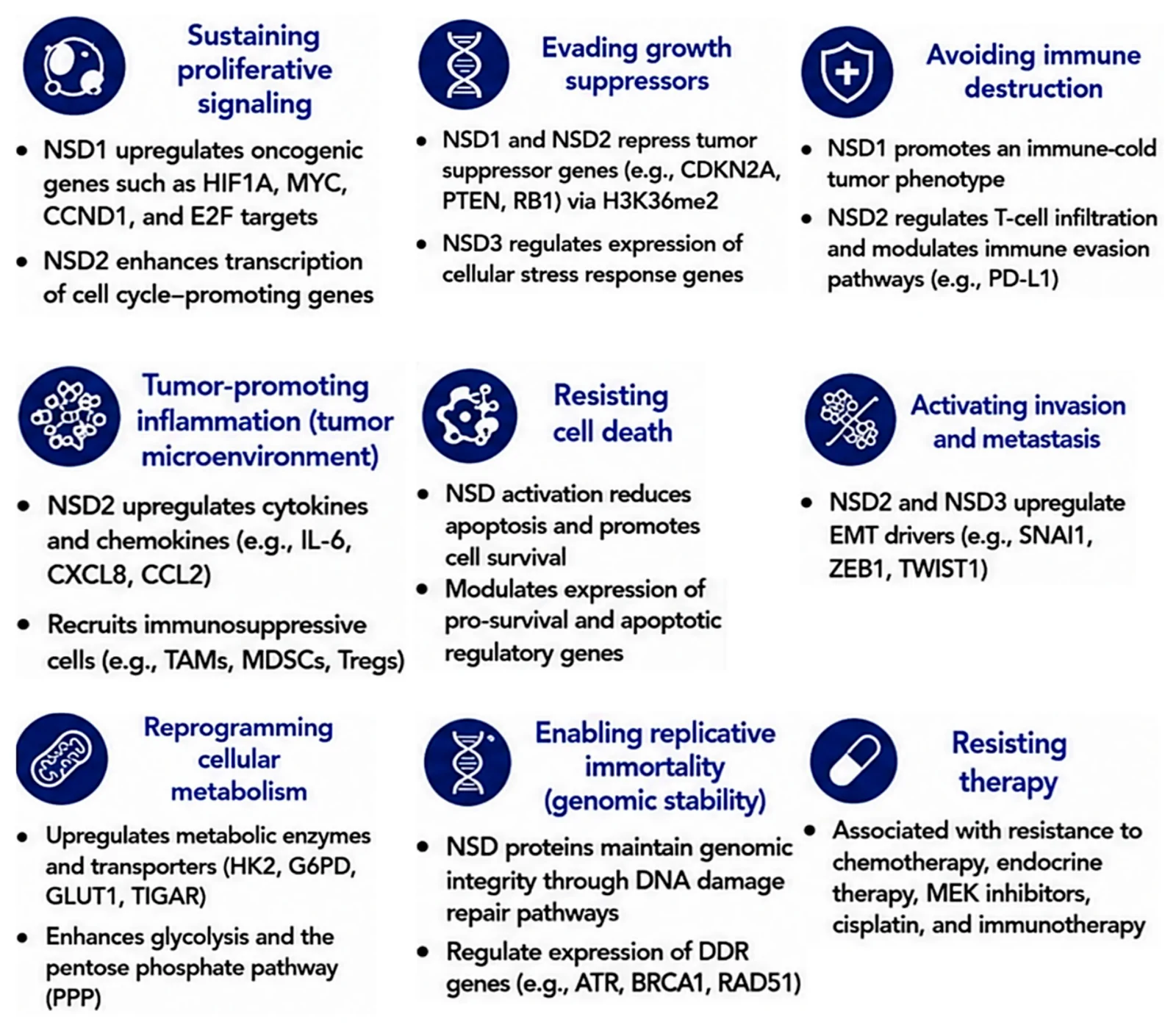
NSD proteins regulate multiple hallmarks of solid tumor cancer. Created in BioRender. Plenn, A. (2026) https://BioRender.com/6i8vo5d (accessed on 30 August 2026).

Although some key functions of NSD family proteins were found in hematologic tumors in the early period of research into the NSD family, the gene alteration frequency is much higher in solid tumors (Figure S1), and accumulating evidence has established the NSD family as critical regulators of solid tumor biology in recent years (Tables S1 and S2). Through their ability to modify chromatin structure and regulate transcriptional programs, NSD proteins influence many of the hallmarks of cancer (Figure 2), including uncontrolled proliferation, invasion, metastatic dissemination, immune evasion and therapeutic resistance. Dysregulation of NSD family members through mutation, amplification, translocation, epigenetic silence, or aberrant overexpression has been reported across a broad range of malignancies, including head and neck, breast, lung, colorectal, gastric, pancreatic, liver, ovarian, bladder, melanoma, and prostate cancers. Collectively, these observations support a model in which NSD proteins function as master epigenetic regulators that integrate chromatin remodeling with oncogenic signaling pathways to drive tumor initiation, progression, and metastatic dissemination [5,19]. A defining feature of NSD-driven cancers is widespread transcriptional reprogramming. Through deposition of H3K36 methylation marks, NSD proteins alter chromatin accessibility and establish permissive environments for oncogenic gene expression [10,16]. These epigenetic changes influence enhancer activity, transcription factor recruitment, and RNA polymerase II elongation, ultimately promoting transcriptional programs that favor tumor growth and survival [10,16]. Although NSD1, NSD2, and NSD3 share overlapping enzymatic functions, each family member contributes to tumorigenesis through distinct biological mechanisms and displays unique patterns of dysregulation across solid tumors [19].

### 3.1. NSD1 in Solid Tumors

NSD1 exhibits particularly context-dependent functions in solid tumors and has been reported to act as either a tumor suppressor or oncogenic regulator depending on cellular context. Originally identified as a nuclear receptor-interacting protein with characteristics of both transcriptional coactivators and corepressors, NSD1 regulates developmental transcriptional programs and hormone receptor signaling pathways through multiple chromatin-reader domains and transcriptional regulatory motifs. In head and neck squamous cell carcinoma (HNSCC), recurrent mutations and inactivation of NSD1 define a distinct molecular subtype characterized by widespread DNA hypomethylation and profound epigenetic deregulation [20,21]. These findings established NSD1 as a major determinant of epigenetic identity in squamous malignancies and highlighted the importance of chromatin regulation in tumor evolution. Beyond regulating tumor cell transcription, NSD1 has emerged as an important mediator of interactions between tumor cells and the immune microenvironment. Comprehensive genomic analyses demonstrated that NSD1 inactivation defines an immune-cold subtype of squamous cell carcinoma characterized by reduced T-cell infiltration and diminished expression of immune-related genes [21]. These findings suggest that NSD1 functions as a tumor cell-intrinsic regulator of immune exclusion and indicate that disruption of NSD1-dependent chromatin regulation can influence antitumor immune responses. The discovery of this immune-cold phenotype has expanded the biological significance of NSD1 beyond tumor cell proliferation alone and suggests that NSD-mediated epigenetic regulation contributes to shaping the composition and function of the tumor microenvironment.

The biological functions of NSD1 extend beyond squamous malignancies. Epigenetic silence of NSD1 has been reported in neuroblastoma and glioma, where loss of expression is associated with altered histone methylation patterns and disruption of normal chromatin organization [22]. Conversely, elevated NSD1 expression has been linked to disease progression in prostate cancer and melanoma, illustrating the context-dependent nature of NSD1 activity. Bianco-Miotto and colleagues further demonstrated that global histone modification patterns and NSD-associated epigenetic signatures correlate with prostate cancer progression, supporting a broader role for NSD1-mediated chromatin regulation in aggressive disease behavior [23]. NSD1 has been implicated in endocrine therapy resistance, including resistance to tamoxifen in breast cancer models [24]. In resistant settings, NSD1 knockdown reduces cell viability and induces apoptosis through disruption of a regulatory axis involving H3K27me3 balance and Wnt/β-catenin signaling [25]. NSD1 has also been associated with increased migration and invasion through NF-κB–linked transcriptional regulation and FBXL11-associated pathways [26]. Additionally, structural alterations such as rearrangements and truncations of NSD1 have been observed in specific breast cancer cell lines, suggesting a contribution to tumor heterogeneity [27]. Together, these observations indicate that NSD1 functions as a critical regulator of epigenetic homeostasis whose disruption can contribute to tumorigenesis through diverse mechanisms depending on tissue context.

### 3.2. NSD2 in Solid Tumor

NSD2 has emerged as the most consistently oncogenic and extensively studied regulator of solid tumor progression. NSD2 overexpression promotes widespread accumulation of H3K36me2, resulting in large-scale chromatin remodeling and activation of oncogenic transcriptional programs [28,29]. Elevated NSD2 expression has been reported in breast, prostate, colorectal, gastric, pancreatic, bladder, skin, and lung cancers, where increased expression frequently correlates with advanced disease stage, metastatic progression, and poor clinical outcome. Unlike NSD1, whose functions may vary across tumor types, NSD2 generally exhibits tumor-promoting activity and is increasingly recognized as a central driver of aggressive cancer phenotypes. Functional studies have demonstrated that NSD2 contributes directly to tumor growth, invasion, and metastatic dissemination.

NSD2 is the most extensively characterized NSD family member in prostate cancer, and acts as a chromatin co-regulator that supports AR-driven transcriptional activity [30]. NSD2 activates TWIST1-dependent transcriptional programs that promote epithelial–mesenchymal transition (EMT), migration, invasion, and acquisition of metastatic characteristics. NSD2 protein regulates T-cell infiltration in prostate cancer, suggesting its potential as a target to enhance immunotherapy effectiveness [31]. NSD2 is frequently overexpressed in breast cancer and functions as an epigenetic co-regulator that supports transcriptional reprogramming in hormone-responsive and aggressive tumor subtypes. NSD2 has been shown to associate with estrogen receptor α (ERα)-linked transcriptional complexes through interactions involving chromatin-associated factors such as BRD proteins, contributing to enhanced ER signaling and endocrine therapy resistance, including tamoxifen resistance [32]. In more aggressive disease states, NSD2 has also been linked to activation of receptor tyrosine kinase signaling programs, including EGFR-AKT axis regulation through transcriptional upregulation of EGFR pathway components [33]. In parallel, NSD2 contributes to transcriptional activation of Wnt/β-catenin signaling through regulation of upstream transcription factors such as FOXM1 and modulation of β-catenin localization [34]. Additionally, NSD2 has been associated with metabolic reprogramming, including regulation of glycolysis and pentose phosphate pathway genes such as HK2, G6PD, GLUT1, and TIGAR [35]. In lung cancer, NSD2 has been associated with transcriptional amplification programs, particularly in KRAS-driven contexts. By increasing H3K36me2 deposition across oncogenic regulatory regions, NSD2 is linked to enhanced tumor growth and poorer clinical outcomes. Importantly, NSD2 depletion has been shown to increase sensitivity to MEK inhibition, suggesting that NSD2 contributes to MAPK pathway dependency through epigenetic reinforcement of oncogenic transcriptional programs [36]. Elevated NSD2 expression has also been observed in gastrointestinal tract cancers, where increased expression is associated with enhanced tumor growth and poor prognosis. In colorectal cancer, NSD2 expression is significantly increased relative to normal tissues and contributes to malignant progression through activating growth and survival pathways including EGFR, MET, SOX2, and ADAM9, along with PI3K/AKT signaling, and genetic depletion reduces tumor growth in vivo [37,38]. In esophageal squamous cell carcinoma, NSD2 contributes to cisplatin resistance through regulation of the MACC1-AS1 axis, with knockdown restoring chemosensitivity in vivo models [39]. In gastric cancer, NSD2 expression correlates with chemotherapy response and prognostic outcomes [40]. In renal cell carcinoma, NSD2 promotes EMT-associated transcriptional programs linked to increased migration and invasion [41]. In melanoma, NSD2 has been associated with therapy-induced dedifferentiation and adaptive resistance mechanisms involving post-transcriptional regulation [42]. Collectively, these findings identify NSD2 as a critical regulator of tumor progression by integrating hormone signaling, growth factor pathways, and metabolic adaptation rather than acting through a single linear pathway.

### 3.3. NSD3 in Solid Tumor

NSD3 has similarly emerged as an important oncogenic regulator in solid tumors. Located within the frequently amplified 8p11-p12 chromosomal region, NSD3 is recurrently amplified in many solid tumors. Amplification of this locus is associated with increased tumor aggressiveness and poor clinical outcome, underscoring the importance of NSD3-driven transcriptional programs in cancer progression. Unlike NSD1 and NSD2, NSD3 contributes to tumorigenesis through both catalytic and non-catalytic mechanisms, reflecting the distinct biological functions of its long and short isoforms [43]. Mechanistically, NSD3 promotes oncogenesis through regulation of transcriptional programs that govern proliferation, survival, and cellular adaptation. The short isoform, NSD3S, functions as a transcriptional adaptor that interacts with BRD4 and CHD8 to drive MYC-dependent transcriptional programs [44]. Disruption of this complex suppresses MYC expression and impairs tumor cell proliferation, demonstrating the importance of NSD3-mediated transcriptional regulation in malignant growth [44]. NSD3S additionally contributes to replication fork protection and maintenance of genome stability, providing a mechanism through which NSD3 can support tumor cell survival during replication stress [43,45]. In contrast, the catalytic NSD3L isoform promotes oncogenic transcription through H3K36 methylation-dependent mechanisms and cooperates with EZH2 and RNA polymerase II to activate signaling pathways associated with invasion and progression [46].

NSD3 is amplified in breast cancer cell lines and primary tumors, supporting its oncogenic relevance [8]. Functional studies have shown that NSD3 depletion affects proliferation and invasive capacity in breast cancer cells [47], while in vivo models suggest that NSD3 overexpression can promote mammary epithelial hyperplasia and tumor-like phenotypes [48]. Mechanistically, NSD3 has been linked to activation of Notch signaling programs through H3K36me2-associated chromatin remodeling, contributing to epithelial-to-mesenchymal transition (EMT), reduced epithelial marker expression such as E-cadherin, and expansion of tumor-initiating cell populations [46]. NSD3 is frequently amplified in lung squamous cell carcinoma and contributes to transcriptional activation of growth and survival pathways, including MYC- and mTOR-associated programs through chromatin remodeling at amplified loci [49]. Within the 8p11 amplicon, NSD3 may act as a functionally relevant driver influencing tumor progression [50]. In addition, NSD3 has been linked to STAT3-associated transcriptional regulation and metabolic gene expression such as Hexokinase 2 (HK2) in certain contexts [51]. Chemical probe studies targeting the NSD3 PWWP1 domain further support its functional importance and suggest potential therapeutic vulnerability [52,53].

**Figure 3:**
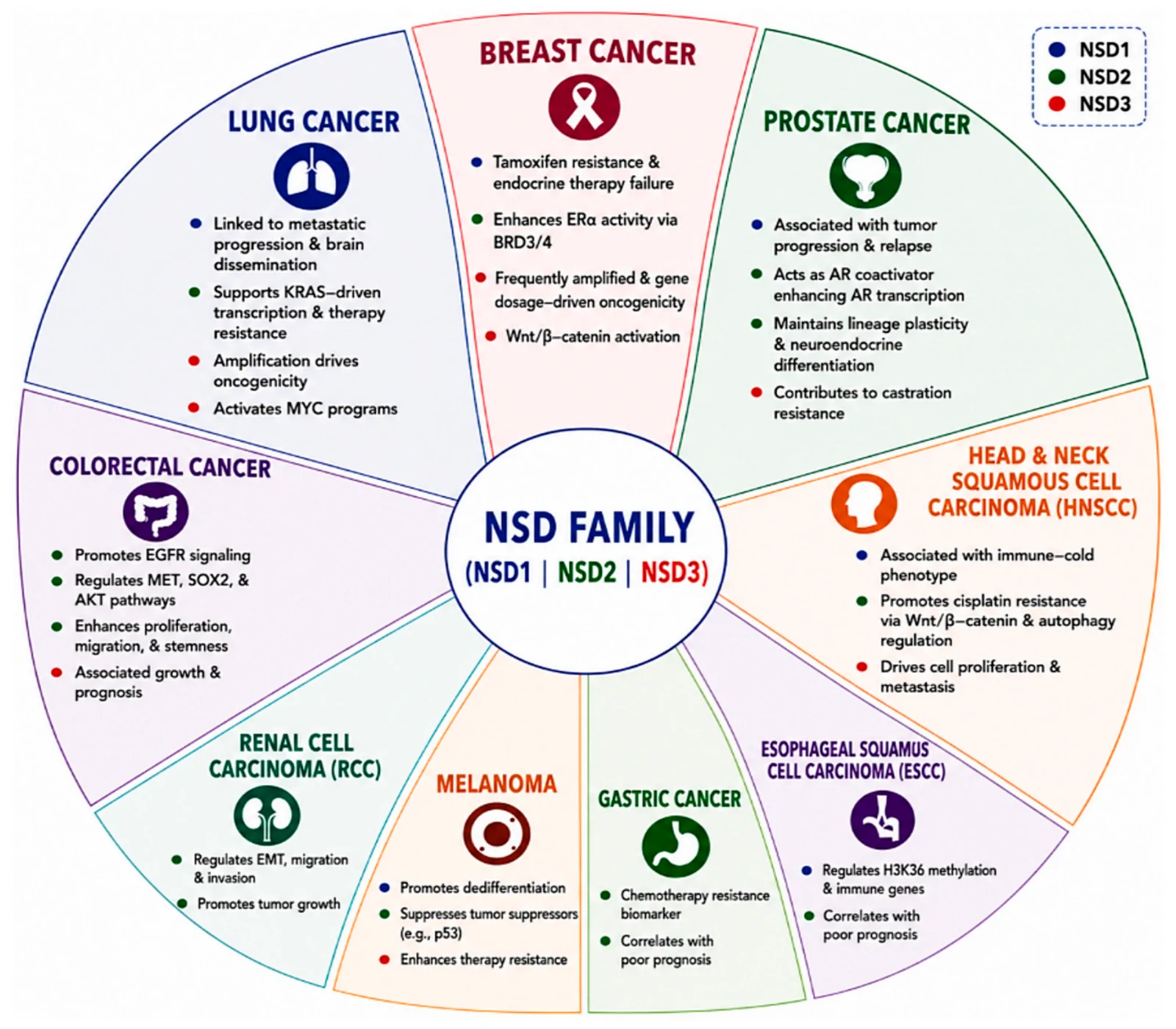
Summary of the roles of NSD family proteins based on solid tumor type. Created in BioRender. Plenn, A. (2026) https://BioRender.com/gf94yoq (accessed on 30 August 2026).

Collectively, the available evidence demonstrates that NSD proteins function as important drivers of solid tumor biology (Figure 3). Through regulation of proliferation, invasion, metastasis, immune evasion, lineage plasticity, and therapeutic adaptation, NSD family members influence nearly every stage of tumor progression. Although the precise molecular mechanisms vary between NSD family members, recurring themes including chromatin reprogramming, metastatic dissemination, immune modulation, and treatment resistance highlight their shared importance in oncogenesis. These biological insights provide a strong rationale for the growing interest in NSD-directed therapeutic strategies and establish the foundation for recent advances in therapeutic targeting and clinical translation.

## 4. Therapeutic Targeting of NSD Proteins and Clinical Advances

The recognition of NSD family proteins as key epigenetic drivers of tumor progression has stimulated considerable interest in their therapeutic targeting. Although NSD proteins were once considered difficult drug targets because of their large multidomain structures and the conserved nature of SET-domain methyltransferases, advances in structural biology, medicinal chemistry, and targeted protein degradation have transformed this view. Cryo-electron microscopy studies revealing nucleosome-dependent activation of NSD2 and NSD3 provided important mechanistic insights and exposed therapeutically exploitable vulnerabilities within these enzymes [4]. Together, these discoveries established the NSD family as an increasingly tractable class of epigenetic targets and laid the foundation for the rapid development of selective inhibitors, chromatin-reader antagonists, and targeted degraders.

Initial drug discovery efforts focused primarily on establishing whether NSD proteins could be pharmacologically inhibited. Early proof-of-concept studies identified mitoxantrone, quinacrine, and chloroquine as compounds capable of disrupting interactions between the NSD1 PHD finger and NIZP1 [54]. Although these molecules were not further developed as selective NSD1 inhibitors, they established proof-of-concept that non-catalytic regulatory surfaces of NSD proteins can be pharmacologically targeted. Plenty of drug discovery efforts concentrated on NSD2 [55], the most extensively investigated and therapeutically advanced NSD family member. Inhibitors such as LEM-06 [56] and PTD2 [57] exhibited modest potency but demonstrated that NSD2 methyltransferase activity could be directly inhibited. Together, these findings demonstrated that NSD2-driven epigenetic programs could be pharmacologically disrupted, validating catalytic inhibition as a viable therapeutic strategy. As inhibitor potency improved, meaningful antitumor effects became evident. MCTP-39 provided some of the first evidence linking NSD2 inhibition to suppression of aggressive cancer phenotypes. In metastatic prostate cancer models, MCTP-39 treatment impaired clonogenic growth, migration, and invasion while disrupting transcriptional programs associated with metastasis. Importantly, these effects translated into significant in vivo activity, including reduced tumor growth, lower H3K36me2 levels, and diminished metastatic burden [58].

A deeper understanding of NSD biology subsequently revealed that therapeutic benefit could be achieved without directly inhibiting catalytic activity. Recognition of the PWWP chromatin-reader domain as a critical mediator of chromatin engagement shifted attention toward disrupting NSD recruitment rather than methyltransferase function itself. This alternative strategy demonstrated that interference with chromatin recognition alone was sufficient to impair NSD-dependent transcriptional programs. Several compounds have validated this approach, such as MR837 [59], Compound 38 [60], and UNC6934 [61]. This strategy produced striking biological effects. Compound 38 reduced proliferation, induced apoptosis, and promoted cell-cycle arrest while suppressing oncogenic transcriptional programs without major changes in global H3K36me2 levels [60]. Similarly, UNC6934 displaced NSD2 from chromatin, disrupted interactions with H3K36me2-marked nucleosomes, and altered subcellular localization of the protein [61]. These studies demonstrated that interference with chromatin recruitment may represent an alternative therapeutic strategy to direct catalytic inhibition. A similar evolution has occurred in the development of NSD3-directed therapeutics. Early work focused on disrupting chromatin recruitment using BI-9321, the first selective NSD3-PWWP1 antagonist. By displacing NSD3 from chromatin, BI-9321 suppressed NSD3-dependent transcriptional programs and provided proof-of-principle that interference with chromatin engagement alone could impair oncogenic NSD3 signaling [52]. More recently, medicinal chemistry efforts have successfully targeted the NSD3 SET domain. Among these compounds, compound 13i represents a notable milestone because structural studies revealed a unique bivalent binding mode that simultaneously engaged both the SAM-binding pocket and an adjacent auxiliary cavity within the SET domain [62]. This compound reduced H3K36me2 and H3K36me3 levels while suppressing proliferation of NSD3-overexpressing breast cancer cells [62]. Additional covalent NSD3 inhibitors including BT5 [63], SZ881, SZ1284, and SLN479 [64], have further expanded the range of NSD3-targeted compounds.

The emergence of targeted protein degradation has accelerated therapeutic development of targeting NSD proteins. Unlike conventional inhibitors, degraders eliminate the entire protein, thereby suppressing both enzymatic and scaffold-dependent functions. Early degraders such as MS159 demonstrated the feasibility of this approach by selectively reducing NSD2 abundance and lowering H3K36me2 levels [65]. Subsequent compounds including UNC8153 and UNC8732 improved degradation efficiency and target engagement [66]. LLC0424 represents one of the most notable examples. This degrader achieved near-complete depletion of NSD2 with a DC50 of approximately 20 nM and a maximal degradation efficiency approaching 96%. In cellular models, LLC0424 induced rapid degradation of both NSD2 isoforms, reduced H3K36me2 levels, suppressed proliferation and colony formation, and altered leukemia-associated transcriptional programs. Importantly, administration in xenograft models resulted in robust degradation of NSD2 protein in vivo, demonstrating effective target engagement and providing compelling evidence that degradation-based strategies may overcome limitations associated with catalytic inhibition alone [67]. Comparable progress has been achieved in targeting NSD3. The first selective NSD3 degrader, MS9715, induced degradation of both long and short NSD3 isoforms, reduced c-MYC expression, promoted apoptosis, suppressed colony formation, and inhibited growth [68]. This approach is particularly attractive because emerging evidence indicates that the short NSD3 isoform (NSD3S) retains potent oncogenic activity despite lacking methyltransferase function [43]. MS9715 mainly demonstrates its effectiveness in hematological malignancies, and it is worth further testing its antitumor effects in solid tumors. Generally, degraders may provide broader therapeutic activity than catalytic inhibitors by simultaneously eliminating both enzymatic and scaffold-dependent functions.

An important consideration for the clinical development of NSD-targeted therapies is the potential for on-target toxicity because NSD proteins contribute to normal development, differentiation, and tissue homeostasis. However, emerging pharmacological evidence suggests that a therapeutically exploitable window may exist, particularly for NSD2 inhibition. The selective NSD2 inhibitors IACS-17596 and IACS-17817 exhibit low- to single-digit nanomolar potency against NSD2 and produced substantial antitumor activity and on-target reduction in H3K36me2 in vivo while maintaining body weight, normal blood counts and serum chemistry, and without apparent structural or functional abnormalities in treated mice [6]. Structural investigations revealed that these compounds competitively bind the SAM-binding pocket and inhibit catalysis through a binary-channel obstruction mechanism. In vitro treatment reduced H3K36me2 levels, rewired chromatin accessibility, and suppressed NSD2-dependent transcriptional programs across multiple cancer models. Importantly, these molecular effects are translated into substantial therapeutic benefits in vivo. In KRAS-driven lung adenocarcinoma and pancreatic ductal adenocarcinoma models, treatment produced marked tumor growth inhibition and reprogrammed oncogenic chromatin states. Similar efficacy was observed in patient-derived xenograft models, where NSD2 inhibition reduced tumor burden and prolonged survival. These findings establish NSD2 as a bona fide epigenetic dependency in solid tumors and strongly support clinical translation of NSD-targeted therapies. Additional translational advances continue to emerge. RK-552 demonstrated potent suppression of NSD2 activity, reducing H3K36me2 and IRF4 expression while inducing apoptosis and suppressing growth of NSD2-dependent cancer cells. Significant reductions in xenograft burden and prolonged survival were observed in vivo, particularly when combined with pomalidomide [69]. Together, these studies highlight the expanding clinical relevance of NSD-directed therapies across diverse malignancies.

**Figure 4:**
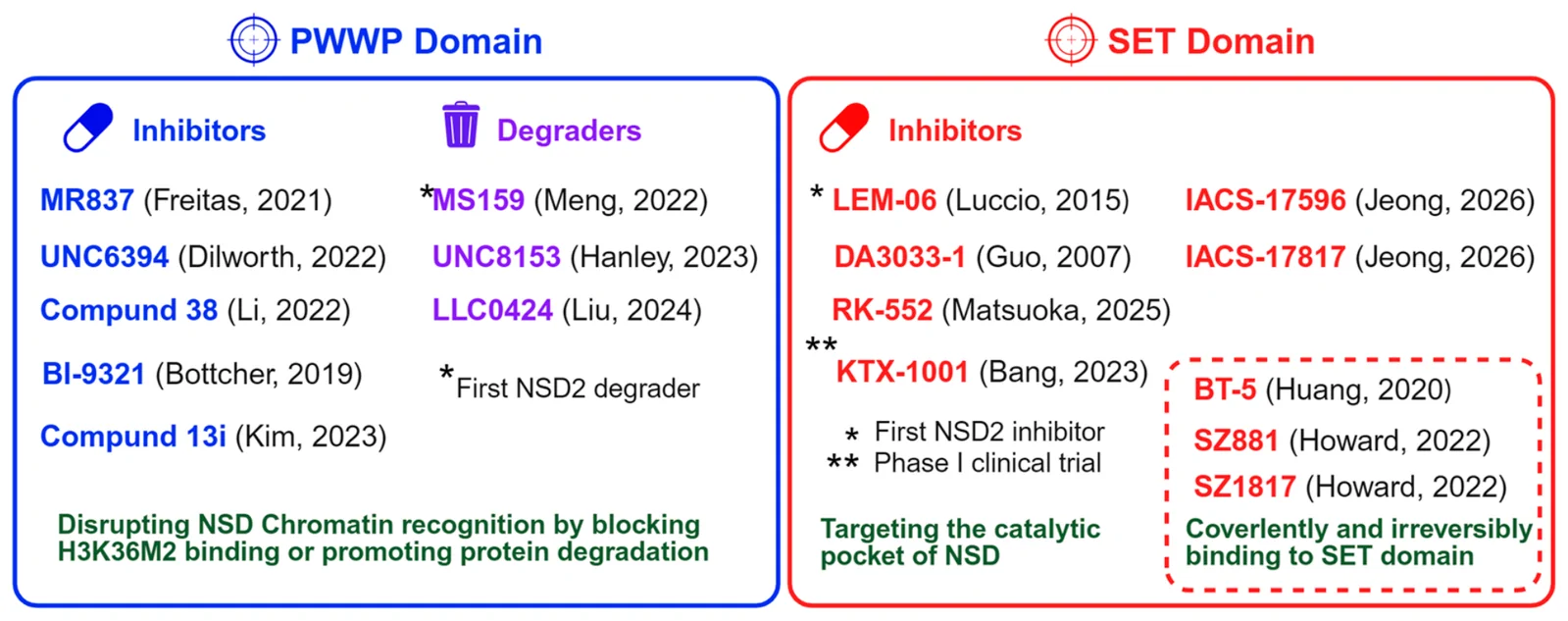
Therapeutic strategies targeting NSD proteins [6,52,56,59,60,61,62,63,64,65,66,67,69,70,71]. Representative compounds targeting PWWP domain and SET domain of NSD2 and NSD3 are shown, including early inhibitors, selective catalytic inhibitors, chromatin-displacing agents, and targeted degraders. Created in BioRender. Song, S. (2026) https://BioRender.com/tx1ov08 (accessed on 4 September 2026).

Despite these advances, several challenges remain. Most currently available inhibitors primarily target catalytic SET-domain functions and therefore predominantly affect full-length NSD proteins. However, accumulating evidence indicates that short isoforms, particularly NSD3S, retain significant oncogenic activity through methyltransferase-independent mechanisms, including BRD4–CHD8-mediated transcriptional regulation and replication fork protection [44,45]. Consequently, catalytic inhibition alone may not fully suppress NSD-driven tumorigenesis. This limitation has increased interest in degraders, PWWP antagonists, and protein–protein interaction inhibitors capable of targeting both catalytic and non-catalytic functions. To minimize adverse effects and maximize therapeutic selectivity, NSD-targeted therapies should incorporate biomarker-guided patient selection based on NSD amplification, overexpression, activating alterations, or functional NSD dependency, together with optimized dosing schedules, development of highly selective catalytic or chromatin-reader inhibitors, and tumor-selective drug delivery strategies. Moreover, targeted protein degradation may provide broader suppression of NSD proteins, including methyltransferase-independent functions, but could potentially produce more sustained target suppression and therefore requires careful assessment of normal-tissue safety. As the field continues to evolve, integration of these complementary strategies may be necessary to achieve durable therapeutic responses. The compounds targeting NSD proteins require further evaluation of pharmacokinetics, pharmacodynamics, efficacy, safety, and therapeutic index before clinical application. Overall, the progression from early proof-of-concept inhibitors to highly potent clinical-grade compounds represents a remarkable achievement and firmly establishes NSD proteins as promising therapeutic targets for future epigenetic therapies in solid tumors (Figure 4).

## 5. Conclusions and Perspectives

NSD1, NSD2, and NSD3 have emerged as important epigenetic regulators of solid tumor development and progression, linking chromatin remodeling to oncogenic transcription, signaling, immune regulation, metastasis, and therapeutic resistance. Although their biological functions are dependent on context, accumulating evidence indicates that dysregulated NSD activity can establish transcriptional states that favor tumor growth and adaptation. NSD2 has demonstrated the most consistent oncogenic activity, whereas NSD1 can function as either a tumor suppressor or tumor-promoting factor depending on cellular and tumor context. NSD3 is particularly complex because its long and short isoforms can promote malignancy through both catalytic and methyltransferase-independent mechanisms.

The growing understanding of NSD structure and function has substantially advanced therapeutic development. NSD proteins, once considered challenging drug targets, are now increasingly tractable through complementary approaches that include SET-domain inhibition, disruption of PWWP-mediated chromatin recruitment, interference with protein–protein interactions, and targeted protein degradation. Preclinical studies with NSD2 and NSD3 inhibitors have demonstrated suppression of H3K36 methylation, oncogenic transcriptional programs, tumor cell proliferation, invasion, and metastatic progression. More recently, potent clinical-grade NSD2 inhibitors, including IACS-17596 and IACS-17817, have provided compelling evidence that NSD2 represents a therapeutically actionable epigenetic dependency in solid tumor models. These advances establish a strong foundation for clinical translation of NSD-directed therapies.

Nevertheless, important challenges remain. Catalytic inhibitors may not adequately suppress methyltransferase-independent functions or oncogenic short isoforms, particularly NSD3S. Targeted degraders may therefore offer an important advantage by eliminating both catalytic and scaffold-dependent functions. Future studies should focus on identifying predictive biomarkers of NSD dependency, defining tumor-specific vulnerabilities, and understanding mechanisms of acquired resistance. Rational combinations of NSD inhibitors or degraders with targeted therapies, chemotherapy, or immune checkpoint blockade may further improve therapeutic efficacy. Ultimately, integrating structural biology, chemical biology, multi-omics profiling, and precision oncology will be critical for translating NSD-targeted strategies into effective and durable treatments for patients with NSD-driven solid tumors.

## Data Availability

No new data were created or analyzed in this study. Data sharing is not applicable to this article.

## Supplementary Materials

The following supporting information can be downloaded at: https://www.mdpi.com/article/10.3390/ijms27188115/s1.

## References

[1] Bennett R.L., Swaroop A., Troche C., Licht J.D. (2017). The Role of Nuclear Receptor-Binding SET Domain Family Histone Lysine Methyltransferases in Cancer. Cold Spring Harb. Perspect. Med..

[2] Chesi M., Nardini E., Lim R.S., Smith K.D., Kuehl W.M., Bergsagel P.L. (1998). The t(4;14) translocation in myeloma dysregulates both FGFR3 and a novel gene, MMSET, resulting in IgH/MMSET hybrid transcripts. Blood.

[3] Qiao Q., Li Y., Chen Z., Wang M., Reinberg D., Xu R.M. (2011). The structure of NSD1 reveals an autoregulatory mechanism underlying histone H3K36 methylation. J. Biol. Chem..

[4] Li W., Tian W., Yuan G., Deng P., Sengupta D., Cheng Z., Cao Y., Ren J., Qin Y., Zhou Y. (2021). Molecular basis of nucleosomal H3K36 methylation by NSD methyltransferases. Nature.

[5] Shrestha A., Kim N., Lee S.J., Jeon Y.H., Song J.J., An H., Cho S.J., Kadayat T.M., Chin J. (2021). Targeting the Nuclear Receptor-Binding SET Domain Family of Histone Lysine Methyltransferases for Cancer Therapy: Recent Progress and Perspectives. J. Med. Chem..

[6] Jeong J., Hausmann S., Dong H., Szczepski K., Flores N.M., Garcia Gonzalez A., Shi L., Lu X., Lempiainen J., Jakab M. (2026). NSD2 inhibitors rewire chromatin to treat lung and pancreatic cancers. Nature.

[7] Huang N., vom Baur E., Garnier J.M., Lerouge T., Vonesch J.L., Lutz Y., Chambon P., Losson R. (1998). Two distinct nuclear receptor interaction domains in NSD1, a novel SET protein that exhibits characteristics of both corepressors and coactivators. EMBO J..

[8] Angrand P.O., Apiou F., Stewart A.F., Dutrillaux B., Losson R., Chambon P. (2001). NSD3, a new SET domain-containing gene, maps to 8p12 and is amplified in human breast cancer cell lines. Genomics.

[9] Rayasam G.V., Wendling O., Angrand P.O., Mark M., Niederreither K., Song L., Lerouge T., Hager G.L., Chambon P., Losson R. (2003). NSD1 is essential for early post-implantation development and has a catalytically active SET domain. EMBO J..

[10] Kuo A.J., Cheung P., Chen K., Zee B.M., Kioi M., Lauring J., Xi Y., Park B.H., Shi X., Garcia B.A. (2011). NSD2 links dimethylation of histone H3 at lysine 36 to oncogenic programming. Mol. Cell.

[11] Sato K., Kumar A., Hamada K., Okada C., Oguni A., Machiyama A., Sakuraba S., Nishizawa T., Nureki O., Kono H. (2021). Structural basis of the regulation of the normal and oncogenic methylation of nucleosomal histone H3 Lys36 by NSD2. Nat. Commun..

[12] Tiukacheva E.A., Vassetzky Y., Razin S.V., Fang D., Ulianov S.V. (2026). Many faces of mammalian NSD methyltransferases. Cell. Mol. Biol. Lett..

[13] Chava S., Wajapeyee N. (2025). NSD proteins in anti-tumor immunity and their therapeutic targeting by protein degraders. Cell. Mol. Life Sci..

[14] Lu T., Jackson M.W., Wang B., Yang M., Chance M.R., Miyagi M., Gudkov A.V., Stark G.R. (2010). Regulation of NF-kappaB by NSD1/FBXL11-dependent reversible lysine methylation of p65. Proc. Natl. Acad. Sci. USA.

[15] Zhang J., Lee Y.R., Dang F., Gan W., Menon A.V., Katon J.M., Hsu C.H., Asara J.M., Tibarewal P., Leslie N.R. (2019). PTEN Methylation by NSD2 Controls Cellular Sensitivity to DNA Damage. Cancer Discov..

[16] Martinez-Garcia E., Popovic R., Min D.J., Sweet S.M., Thomas P.M., Zamdborg L., Heffner A., Will C., Lamy L., Staudt L.M. (2011). The MMSET histone methyl transferase switches global histone methylation and alters gene expression in t(4;14) multiple myeloma cells. Blood.

[17] Hollink I.H., van den Heuvel-Eibrink M.M., Arentsen-Peters S.T., Pratcorona M., Abbas S., Kuipers J.E., van Galen J.F., Beverloo H.B., Sonneveld E., Kaspers G.J. (2011). NUP98/NSD1 characterizes a novel poor prognostic group in acute myeloid leukemia with a distinct HOX gene expression pattern. Blood.

[18] French C.A., Rahman S., Walsh E.M., Kuhnle S., Grayson A.R., Lemieux M.E., Grunfeld N., Rubin B.P., Antonescu C.R., Zhang S. (2014). NSD3-NUT fusion oncoprotein in NUT midline carcinoma: Implications for a novel oncogenic mechanism. Cancer Discov..

[19] Topchu I., Pangeni R.P., Bychkov I., Miller S.A., Izumchenko E., Yu J., Golemis E., Karanicolas J., Boumber Y. (2022). The role of NSD1, NSD2, and NSD3 histone methyltransferases in solid tumors. Cell. Mol. Life Sci..

[20] Farhangdoost N., Horth C., Hu B., Bareke E., Chen X., Li Y., Coradin M., Garcia B.A., Lu C., Majewski J. (2021). Chromatin dysregulation associated with NSD1 mutation in head and neck squamous cell carcinoma. Cell Rep..

[21] Brennan K., Shin J.H., Tay J.K., Prunello M., Gentles A.J., Sunwoo J.B., Gevaert O. (2017). NSD1 inactivation defines an immune cold, DNA hypomethylated subtype in squamous cell carcinoma. Sci. Rep..

[22] Berdasco M., Ropero S., Setien F., Fraga M.F., Lapunzina P., Losson R., Alaminos M., Cheung N.K., Rahman N., Esteller M. (2009). Epigenetic inactivation of the Sotos overgrowth syndrome gene histone methyltransferase NSD1 in human neuroblastoma and glioma. Proc. Natl. Acad. Sci. USA.

[23] Bianco-Miotto T., Chiam K., Buchanan G., Jindal S., Day T.K., Thomas M., Pickering M.A., O’Loughlin M.A., Ryan N.K., Raymond W.A. (2010). Global levels of specific histone modifications and an epigenetic gene signature predict prostate cancer progression and development. Cancer Epidemiol. Biomark. Prev..

[24] Mendes-Pereira A.M., Sims D., Dexter T., Fenwick K., Assiotis I., Kozarewa I., Mitsopoulos C., Hakas J., Zvelebil M., Lord C.J. (2012). Genome-wide functional screen identifies a compendium of genes affecting sensitivity to tamoxifen. Proc. Natl. Acad. Sci. USA.

[25] Chen Y., Li X., Xu J., Xiao H., Tang C., Liang W., Zhu X., Fang Y., Wang H., Shi J. (2022). Knockdown of nuclear receptor binding SET domain-containing protein 1 (NSD1) inhibits proliferation and facilitates apoptosis in paclitaxel-resistant breast cancer cells via inactivating the Wnt/beta-catenin signaling pathway. Bioengineered.

[26] Chen Y., Tang W., Zhu X., Zhang L., Zhu Y., Xiao H., Xu J., Fang Y., Li X., Tang C. (2021). Nuclear receptor binding SET domain protein 1 promotes epithelial-mesenchymal transition in paclitaxel-resistant breast cancer cells via regulating nuclear factor kappa B and F-box and leucine-rich repeat protein 11. Bioengineered.

[27] Zhao Q., Caballero O.L., Levy S., Stevenson B.J., Iseli C., de Souza S.J., Galante P.A., Busam D., Leversha M.A., Chadalavada K. (2009). Transcriptome-guided characterization of genomic rearrangements in a breast cancer cell line. Proc. Natl. Acad. Sci. USA.

[28] Morishita M., di Luccio E. (2011). Cancers and the NSD family of histone lysine methyltransferases. Biochim. Biophys. Acta.

[29] Han X., Piao L., Xu X., Luo F., Liu Z., He X. (2020). NSD2 Promotes Renal Cancer Progression Through Stimulating Akt/Erk Signaling. Cancer Manag Res..

[30] Parolia A., Eyunni S., Verma B.K., Young E., Liu Y., Liu L., George J., Aras S., Das C.K., Mannan R. (2024). NSD2 is a requisite subunit of the AR/FOXA1 neo-enhanceosome in promoting prostate tumorigenesis. Nat. Genet.

[31] Li Q., Zhu J., Zhang Y., Pan Y., Li Z., Wang M., Gao Y., Feng D., He X., Zhang C. (2023). Association of WHSC1/NSD2 and T-cell infiltration with prostate cancer metastasis and prognosis. Sci. Rep..

[32] Feng Q., Zhang Z., Shea M.J., Creighton C.J., Coarfa C., Hilsenbeck S.G., Lanz R., He B., Wang L., Fu X. (2014). An epigenomic approach to therapy for tamoxifen-resistant breast cancer. Cell Res..

[33] Wang J.J., Zou J.X., Wang H., Duan Z.J., Wang H.B., Chen P., Liu P.Q., Xu J.Z., Chen H.W. (2019). Histone methyltransferase NSD2 mediates the survival and invasion of triple-negative breast cancer cells via stimulating ADAM9-EGFR-AKT signaling. Acta Pharmacol. Sin..

[34] Zhang J., Lu J., Chen Y., Li H., Lin L. (2020). WHSC1 promotes wnt/beta-catenin signaling in a FoxM1-dependent manner facilitating proliferation, invasion and epithelial-mesenchymal transition in breast cancer. J. Recept. Signal Transduct. Res..

[35] Wang Q., Zheng J., Zou J.X., Xu J., Han F., Xiang S., Liu P., Chen H.W., Wang J. (2020). S-adenosylhomocysteine (AdoHcy)-dependent methyltransferase inhibitor DZNep overcomes breast cancer tamoxifen resistance via induction of NSD2 degradation and suppression of NSD2-driven redox homeostasis. Chem. Biol. Interact..

[36] Sengupta D., Zeng L., Li Y., Hausmann S., Ghosh D., Yuan G., Nguyen T.N., Lyu R., Caporicci M., Morales Benitez A. (2021). NSD2 dimethylation at H3K36 promotes lung adenocarcinoma pathogenesis. Mol. Cell.

[37] Zhao L.H., Li Q., Huang Z.J., Sun M.X., Lu J.J., Zhang X.H., Li G., Wu F. (2021). Identification of histone methyltransferase NSD2 as an important oncogenic gene in colorectal cancer. Cell Death Dis..

[38] D’Afonseca V., Gonzalez G., Salazar M., Arencibia A.D. (2020). Computational analyses on genetic alterations in the NSD genes family and the implications for colorectal cancer development. Ecancermedicalscience.

[39] Xue W., Shen Z., Li L., Zheng Y., Yan D., Kan Q., Zhao J. (2021). Long non-coding RNAs MACC1-AS1 and FOXD2-AS1 mediate NSD2-induced cisplatin resistance in esophageal squamous cell carcinoma. Mol. Ther. Nucleic Acids.

[40] Wei J., Costa C., Shen J., Yu L., Sanchez J.J., Qian X., Sun X., Zou Z., Gimenez-Capitan A., Yue G. (2014). Differential effect of MMSET mRNA levels on survival to first-line FOLFOX and second-line docetaxel in gastric cancer. Br. J. Cancer.

[41] Han X., Piao L., Yuan X., Wang L., Liu Z., He X. (2019). Knockdown of NSD2 Suppresses Renal Cell Carcinoma Metastasis by Inhibiting Epithelial-Mesenchymal Transition. Int. J. Med. Sci..

[42] De Santis A., De Santis L., Rossi F., Gasparini S., Licursi V., Amico V.A., Capone I., Fragale A., D’Atri S., Gabriele L. (2025). NSD2 and miRNAs as Key Regulators of Melanoma Response to Romidepsin and Interferon-alpha2b Treatment. Cancer Med..

[43] Nunez Y., Vera S., Baeza V., Gonzalez-Pecchi V. (2024). NSD3 in Cancer: Unraveling Methyltransferase-Dependent and Isoform-Specific Functions. Int. J. Mol. Sci..

[44] Shen C., Ipsaro J.J., Shi J., Milazzo J.P., Wang E., Roe J.S., Suzuki Y., Pappin D.J., Joshua-Tor L., Vakoc C.R. (2015). NSD3-Short Is an Adaptor Protein that Couples BRD4 to the CHD8 Chromatin Remodeler. Mol. Cell.

[45] Zhu S., Gao H., Jiang D., Guo G., Hou J., Han Y., Zhang C., Hu X., Indulkar S., Kloeber J.A. (2025). Isoform-specific function of NSD3 in DNA replication stress confers resistance to PARP inhibitors in prostate cancer. Mol. Cell.

[46] Jeong G.Y., Park M.K., Choi H.J., An H.W., Park Y.U., Choi H.J., Park J., Kim H.Y., Son T., Lee H. (2021). NSD3-Induced Methylation of H3K36 Activates NOTCH Signaling to Drive Breast Tumor Initiation and Metastatic Progression. Cancer Res..

[47] Zhou Z., Thomsen R., Kahns S., Nielsen A.L. (2010). The NSD3L histone methyltransferase regulates cell cycle and cell invasion in breast cancer cells. Biochem. Biophys. Res. Commun..

[48] Turner-Ivey B., Smith E.L., Rutkovsky A.C., Spruill L.S., Mills J.N., Ethier S.P. (2017). Development of mammary hyperplasia, dysplasia, and invasive ductal carcinoma in transgenic mice expressing the 8p11 amplicon oncogene NSD3. Breast Cancer Res. Treat..

[49] Yuan G., Flores N.M., Hausmann S., Lofgren S.M., Kharchenko V., Angulo-Ibanez M., Sengupta D., Lu X., Czaban I., Azhibek D. (2021). Elevated NSD3 histone methylation activity drives squamous cell lung cancer. Nature.

[50] Satpathy S., Krug K., Jean Beltran P.M., Savage S.R., Petralia F., Kumar-Sinha C., Dou Y., Reva B., Kane M.H., Avanessian S.C. (2021). A proteogenomic portrait of lung squamous cell carcinoma. Cell.

[51] Zhou Y., Peng X., Fang C., Peng X., Tang J., Wang Z., Long Y., Chen J., Peng Y., Zhang Z. (2024). Histones Methyltransferase NSD3 Inhibits Lung Adenocarcinoma Glycolysis Through Interacting with PPP1CB to Decrease STAT3 Signaling Pathway. Adv. Sci..

[52] Bottcher J., Dilworth D., Reiser U., Neumuller R.A., Schleicher M., Petronczki M., Zeeb M., Mischerikow N., Allali-Hassani A., Szewczyk M.M. (2019). Fragment-based discovery of a chemical probe for the PWWP1 domain of NSD3. Nat. Chem. Biol..

[53] Li Y., Shao S., Lei Q., Song C., Wang S., Deng H. (2026). Fluorogenic Ligand Enables Identification of NSD3-Overexpressed Tumors by Targeting the PWWP1 Domain of NSD3. Anal. Chem..

[54] Berardi A., Ghitti M., Quilici G., Musco G. (2020). In silico derived small molecules targeting the finger-finger interaction between the histone lysine methyltransferase NSD1 and Nizp1 repressor. Comput. Struct. Biotechnol. J..

[55] Ma Z., Bolinger A.A., Chen H., Zhou J. (2023). Drug Discovery Targeting Nuclear Receptor Binding SET Domain Protein 2 (NSD2). J. Med. Chem..

[56] di Luccio E. (2015). Inhibition of Nuclear Receptor Binding SET Domain 2/Multiple Myeloma SET Domain by LEM-06 Implication for Epigenetic Cancer Therapies. J. Cancer Prev..

[57] Morrison M.J., Boriack-Sjodin P.A., Swinger K.K., Wigle T.J., Sadalge D., Kuntz K.W., Scott M.P., Janzen W.P., Chesworth R., Duncan K.W. (2018). Identification of a peptide inhibitor for the histone methyltransferase WHSC1. PLoS ONE.

[58] Aytes A., Giacobbe A., Mitrofanova A., Ruggero K., Cyrta J., Arriaga J., Palomero L., Farran-Matas S., Rubin M.A., Shen M.M. (2018). NSD2 is a conserved driver of metastatic prostate cancer progression. Nat. Commun..

[59] Ferreira de Freitas R., Liu Y., Szewczyk M.M., Mehta N., Li F., McLeod D., Zepeda-Velazquez C., Dilworth D., Hanley R.P., Gibson E. (2021). Discovery of Small-Molecule Antagonists of the PWWP Domain of NSD2. J. Med. Chem..

[60] Li N., Yang H., Liu K., Zhou L., Huang Y., Cao D., Li Y., Sun Y., Yu A., Du Z. (2022). Structure-Based Discovery of a Series of NSD2-PWWP1 Inhibitors. J. Med. Chem..

[61] Dilworth D., Hanley R.P., Ferreira de Freitas R., Allali-Hassani A., Zhou M., Mehta N., Marunde M.R., Ackloo S., Carvalho Machado R.A., Khalili Yazdi A. (2022). A chemical probe targeting the PWWP domain alters NSD2 nucleolar localization. Nat. Chem. Biol..

[62] Kim S., Hwang I., Kim S.H., Chung H.W., Ji M.J., Moon S., Park H.M., Kong G., Hur W. (2023). Identification of novel class inhibitors of NSD3 methyltransferase showing a unique, bivalent binding mode in the SET domain. Chem. Biol. Drug Des..

[63] Huang H., Howard C.A., Zari S., Cho H.J., Shukla S., Li H., Ndoj J., Gonzalez-Alonso P., Nikolaidis C., Abbott J. (2020). Covalent inhibition of NSD1 histone methyltransferase. Nat. Chem. Biol..

[64] Howard C. (2022). Development and Characterization of Irreversible NSD1 And NSD3 Histone Methyltransferase Inhibitors. Ph.D. Thesis.

[65] Meng F., Xu C., Park K.S., Kaniskan H.U., Wang G.G., Jin J. (2022). Discovery of a First-in-Class Degrader for Nuclear Receptor Binding SET Domain Protein 2 (NSD2) and Ikaros/Aiolos. J. Med. Chem..

[66] Hanley R.P., Nie D.Y., Tabor J.R., Li F., Sobh A., Xu C., Barker N.K., Dilworth D., Hajian T., Gibson E. (2023). Discovery of a Potent and Selective Targeted NSD2 Degrader for the Reduction of H3K36me2. J. Am. Chem. Soc..

[67] Liu L., Parolia A., Liu Y., Hou C., He T., Qiao Y., Eyunni S., Luo J., Li C., Wang Y. (2024). Discovery of LLC0424 as a Potent and Selective in Vivo NSD2 PROTAC Degrader. J. Med. Chem..

[68] Xu C., Meng F., Park K.S., Storey A.J., Gong W., Tsai Y.H., Gibson E., Byrum S.D., Li D., Edmondson R.D. (2022). A NSD3-targeted PROTAC suppresses NSD3 and cMyc oncogenic nodes in cancer cells. Cell Chem. Biol..

[69] Matsuoka S., Osada N., Kubota H., Kikuzato K., Koyama H., Sonoda T., Idei A., Yoshida M., Kikuchi M., Umehara T. (2025). Discovery of a novel class NSD2 inhibitor for multiple myeloma with t(4;14)^+^. Blood Neoplasia.

[70] Guo J., Parise R.A., Joseph E., Lan J., Pan S.S., Joo B., Egorin M.J., Wipf P., Lazo J.S., Eiseman J.L. (2007). Pharmacology and antitumor activity of a quinolinedione Cdc25 phosphatase inhibitor DA3003-1 (NSC 663284). Anticancer Res..

[71] ClinicalTrials.gov A Study of an MMSET Inhibitor in Patients with Relapsed and Refractory Multiple Myeloma (NCT05651932). NCT05651932.

