# Supplementary material for: NSD Histone Methyltransferases in Solid Tumors: Biological Functions, Oncogenic Mechanisms and Therapeutic Targeting
Source: Int J Mol Sci. 2026 Sep 12;27(18):8115. doi: 10.3390/ijms27188115 (PMC13607714; doi:10.3390/ijms27188115)
Supplement: Supplementary file 1 [file ijms-27-08115-s001.zip › ijms-4468459-supplementary.pdf]

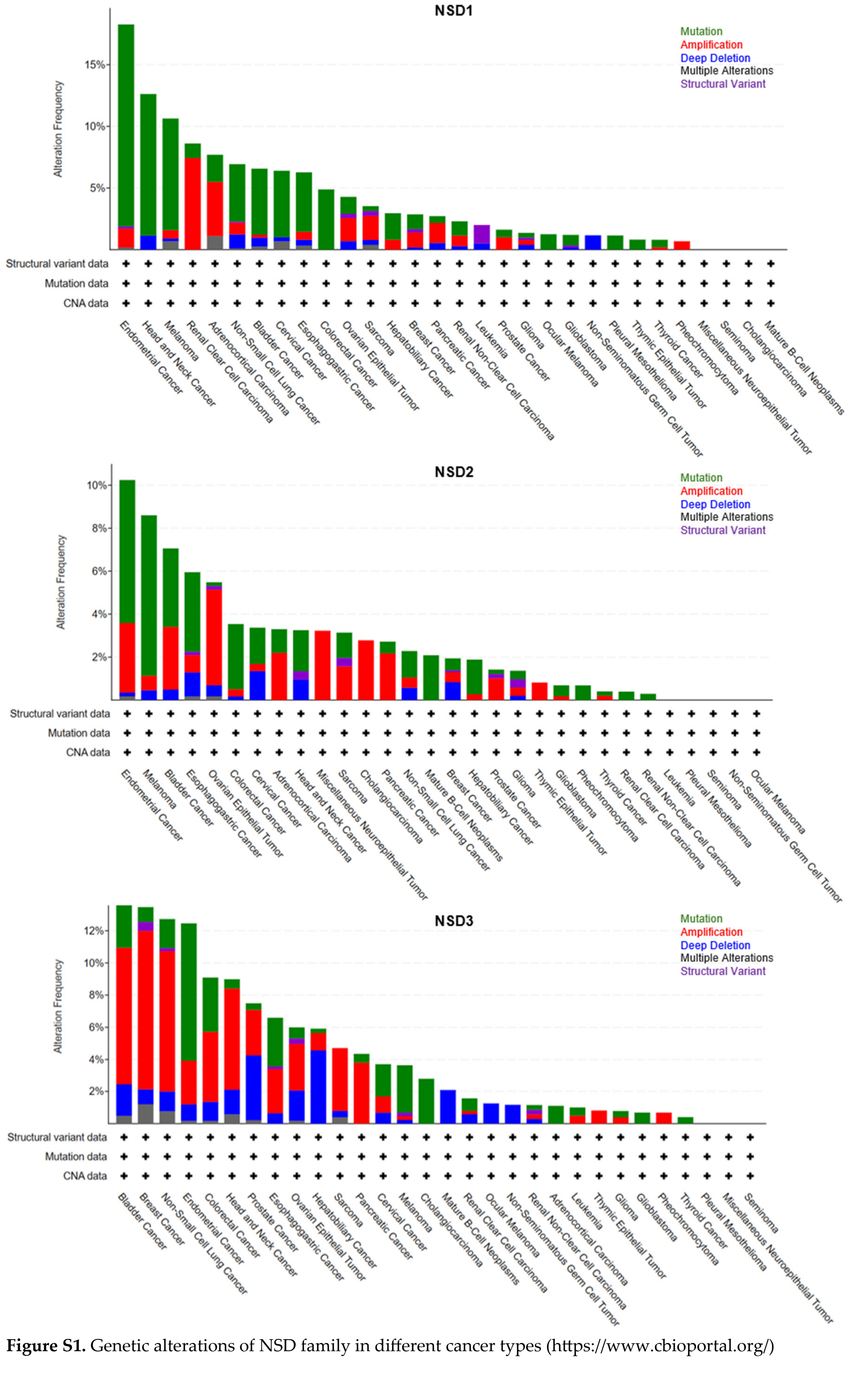

Figure S1. Genetic alterations of NSD family in different cancer types (https://www.cbioportal.org/)

Feature	NSD1	NSD2	NSD3
**Chromosomal location**	5q35.2–q35.3	4p16.3	8p11.23
**Major enzymatic activity**	H3K36me1/2 methyltransferase	H3K36me1/2 methyltransferase	H3K36me1/2 methyltransferase
**Major mechanisms of dysregulation**	Mutation, deletion, epigenetic silence, altered expression	Overexpression, activating mutation, translocation, altered expression	Amplification, activating mutation, altered expression, structural alterations
**Major regulatory mechanisms**	Chromatin-reader interactions, transcriptional regulation, PHD/PWWP-dependent chromatin engagement	Chromatin recruitment through PWWP domains; transcription-factor and signaling interactions	PWWP/SET-dependent chromatin regulation; protein–protein interactions; isoform-specific regulation
**Representative cancer associations**	HNSCC, endometrial, prostate, melanoma, breast, neuroblastoma, glioma	Prostate, breast, lung, colorectal, gastric, pancreatic, bladder, renal cancers	Breast, lung, HNSCC, colorectal, bladder and other 8p11-amplified tumors
**Predominant oncogenic/tumor-suppressive role**	Context dependent; tumor suppressive or oncogenic	Predominantly oncogenic	Predominantly oncogenic
**Major oncogenic functions**	Epigenetic identity, transcriptional regulation, immune modulation, therapy resistance	Proliferation, EMT, metastasis, hormone signaling, metabolic reprogramming, therapy resistance	MYC activation, proliferation, EMT, genome stability, transcriptional regulation
**Representative therapeutic strategies**	PHD/PWWP/SET-domain targeting inhibitors	SET-domain inhibitors, PWWP antagonists, degraders	PWWP inhibitors, SET-domain inhibitors, covalent inhibitors, degraders
**Developmental status**	Predominantly preclinical	Most therapeutically advanced; early clinical evaluation of selected inhibitors	Predominantly preclinical
**Key consideration**	Strong context dependence; NSD1 loss may have tumor-suppressive implications	Strongest evidence for oncogenic dependency and pharmacological targeting	Both catalytic and non-catalytic/isoform-specific functions should be considered

Table S1. Comparison of NSD1, NSD2, and NSD3 in cancer biology and therapeutic targeting.

Tumor type, NSDs	Mechanism/Conclusion	Targeting Strategy, In Vivo test	PMID, Publication year
BC, NSD3	Identified NSD3 and showed it is located on 8p12 and amplified in Human Breast Cancer	N/A, No	11374904, 2001
BC, NSD1	Identified genomic rearrangement of NSD1 in a breast cancer cell line HCC1954	N/A, No	19181860, 2009
BC, NSD3	NSD3L regulates cell cycle and cell invasion and is a candidate tumor suppressor	siRNA, No	20599755, 2010
BC, NSD1	NSD1 gene silencing causes tamoxifen resistance	siRNA, No	21482774, 2011
BC, NSD2	NSD2 is recruited to the Erα gene by BRD3/4 and facilitates ERα gene expression.	siRNA, No	24874954, 2014
BC, NSD3	Amplification of NSD3 regulates expression and estrogen independent activation of ERa in SUM-44 breast cancer cells and is associated with ERa over-expression in breast cancer	siRNA, No	27005559, 2016
BC, NSD2	Reprogramming metabolism by NSD2 drives endocrine resistance via coordinated activation of pentose phosphate pathway enzymes	N/A, No	27164560, 2016
BC, NSD3	NSD3 is a driving breast cancer oncogene from the 8p11 amplicon.	N/A, No	28484924, 2017
BC, NSD2	NSD2 mediates the survival and invasion of TNBC cells via stimulating ADAM9-EGFR-AKT signaling	shRNA, Yes	30670815, 2019
BC, NSD2	DZNep overcomes breast cancer tamoxifen resistance via induction of NSD2 degradation and suppression of NSD2-driven redox homeostasis	DZNep, Yes	32001260, 2020
BC&LC, NSD1	SET7 inhibitor DC21 has good selectivity for NSD1	DC21, No	32173197, 2020
BC, NSD2	NSD2 promotes Wnt/b-catenin signaling in a FoxM1-dependent manner facilitating proliferation, invasion and EMT	shRNA, No	32314642, 2020
BC, NSD3	NSD3 cooperates with EZH2 to activate NOTCH signaling, promoting EMT and breast cancer progression	shRNA, Yes	32967925, 2021
BC, NSD2	Associated with aggressive TNBC and poor prognosis through epigenetic cooperation with EZH2.	shRNA, No	33665162, 2021
BC, NSD1	NSD1 promotes EMT in paclitaxel-resistant BC cells via regulating NFκB and FBXL11	shRNA, Yes	34905470, 2021
BC, NSD1	NSD1 knockdown inhibits proliferation and promotes the apoptosis of paclitaxel-resistant BC cells by inactivating the NSD1/H3K27me3/Wnt10b/β-catenin signaling pathway.	shRNA, Yes	35200072, 2022
BC, NSD3	13i shows a unique, bivalent binding mode interacting with both SAM-binding site and BT3-bindig site within the SET domain	13i, No	37072259, 2023
BC&LC, NSD3	Fluorogenic probe LY-06 enables identification of NSD3-overexpressed tumors by targeting the PWWP1 domain of NSD3	LY-06, Yes	41820229, 2026
LC&PDAC, NSD3	NSD3 is one of the common drivers of the 8p11-12 Amplicon in LC&PDAC	shRNA, Yes	24051013, 2013
LC, NSD3	NSD3 as a principal 8p11–12 amplicon-associated oncogenic driver in LUSC. NSD3-dependency renders LUSC therapeutically vulnerable to bromodomain inhibition.	CRISPR/Cas9, Yes	33536620, 2021
LC, NSD3	NSD3 is an alternative driver in FGFR1-amplified lung squamous cell carcinoma and serves as one of potential therapeutic vulnerabilities	N/A, Yes	34358469, 2021
LC, NSD2	NSD2 maintains H3K36me2-dependent KRAS transcriptional programs, promoting tumor growth and resistance to MEK inhibition.	CRISPR/Cas9,Yes	34555356, 2021
LC, NSD3	NSD3 inhibits LC glycolysis through interacting with PPP1CB to decrease STAT3 Signaling Pathway	CRISPR/Cas9,Yes	39119928, 2024
LC, NSD2	Establishing NSD2 as a promising therapeutic target and supporting combined NSD2–KRAS inhibitor therapy	IACS-17596& 17817, Yes	40770093, 2026
LC, NSD1	NSD1 mutation is among the most frequent alterations in brain metastasis of NSCLC	N/A, No	41248618, 2025
PC, NSD1	Acts as androgen receptor coactivator enhancing AR transcription	N/A, No	11501969, 2001
PC, NSD1	Associated with PC progression and relapse through epigenetic changes.	N/A, No	20841388, 2010
PC, NSD1&3	Contribute to castration resistance via transcriptional control	N/A, No	30616239, 2019
PC, NSD1	Bisphenol A down-regulates NSD1 and alters epigenetic regulation	N/A, No	31610501, 2019
PC, NSD2	NSD2 is key regulator of tumor-resident immune pathways. NSD2 inhibition has the potential to act as a potent adjuvant for combination immunotherapy	MCTP39, Yes	33589522, 2021
PC, NSD2	Maintains lineage plasticity and neuroendocrine differentiation through H3K36me2 mediated androgen receptor enhancer remodeling, promoting resistance to enzalutamide	CRISPR/Cas9,Yes	37502956, 2023
PC, NSD2	NSD2 is associated with an immunosuppressive microenvironment and can be an independent prognostic factor for PC	N/A,NO	38062230, 2023
PC, NSD1&2	NSD2 is a subunit of the AR neo-enhanceosome that wires gene expression programs, positioning NSD1/2 as viable paralog co-targets	LLC0150, Yes	39251788, 2024
GC, NSD2	NSD2 mainly serves as a biomarker for chemotherapy response	N/A, No	24809779, 2014
ESCC, NSD2	Long non-coding RNAs MACC1-AS1 and FOXD2-AS1 mediate NSD2-induced cisplatin resistance in ESCC	shRNA, Yes	33552680, 2020
RCC, NSD2	Down-regulation of NSD2 suppress cell migration and invasion through inhibiting EMT	siRNA, No	31692936, 2019
M, NSD2	NSD2 downregulation following Romidepsin and Interferon-α2b treatment correlating with cellular de-differentiation, which is mediated by miR-23a, miR-24, and miR-31	N/A, No	40386826, 2025
CRC, NSD2	NSD2 exerted oncogenic functions in CRC and could be a promising therapeutic target	shRNA, Yes	34671018, 2021
HNSC&LC, NSD1	NSD1 inactivation is associated with widespread impairment of epigenetic regulation in both HNSC and LUSC. In HNSC, NSD1 inactivation decreases immune cell infiltration	shRNA, Yes	29213088, 2017
GBM, NSD2	NSD2 is a proliferation-related protein in human gliomas	siRNAs, No	18182627,2008
**BC:** Breast Cancer; **LC:** Lung Cancer; **PDAC**: Pancreatic Ductal Adenocarcinoma; **CRC:** Colorectal Cancer; **PC**: Prostate Cancer; **RCC:** Renal Cell Carcinoma; **HNSC:** Head & Neck Squamous Cell Carcinoma; **GA:** Gastric Cancer; **ESCC:** Esophageal Squamous Cell Carcinoma; **M:** Melanoma; **GBM:** Glioblastoma			

Table S2. Overview NSD proteins in solid tumor research.
